# Coeliac artery avulsion secondary to high velocity blunt abdominal trauma: a case report

**DOI:** 10.1093/jscr/rjad615

**Published:** 2023-11-12

**Authors:** Tegan J Kay, Zafreen Rahman, Korana Musicki, Noel Atkinson, Katherine Martin

**Affiliations:** Department of General Surgery, 300 Grattan Street, Parkville, Victoria 3052, Australia; Department of Vascular Surgery, 300 Grattan Street, Parkville, Victoria 3052, Australia; Department of Vascular Surgery, 300 Grattan Street, Parkville, Victoria 3052, Australia; Department of Vascular Surgery, 300 Grattan Street, Parkville, Victoria 3052, Australia; Department of General Surgery, 300 Grattan Street, Parkville, Victoria 3052, Australia

**Keywords:** trauma, vascular, coliac artery, avulsion

## Abstract

Coeliac artery (CA) injuries are an extremely rare subset of blunt abdominal trauma with a reported incidence of only 0.01%. Patterns of CA injury include intimal tear, dissection, thrombosis and pseudoaneurysm, with the most rare being complete CA avulsion. These complex injuries pose a treatment challenge due to rapid blood loss and anatomical difficultly in achieving proximal and multiple points of distal vascular control. To our knowledge, this case of CA avulsion from blunt polytrauma is only the 7th case reported in the literature. To assist in management, we report a case of blunt traumatic CA avulsion managed successfully with open ligation following endovascular balloon occlusion of the juxta-coeliac aorta for haemorrhage control.

## Introduction

Major abdominal visceral vessel injuries are rare and occur in only 5.9% of blunt polytrauma [1]. Patients often present in haemorrhagic shock with resulting mortality as high as 50% [2]. These complex injuries pose a treatment challenge due to rapid blood loss and anatomical difficultly navigating at the depths of the junction of cavities to achieve proximal and multiple points of distal vascular control [3]. Coeliac artery (CA) injuries are an extremely rare subset of blunt abdominal trauma with an incidence of 0.01% [3]. Patterns of CA injury include intimal tear, dissection, thrombosis, and pseudoaneurysm, with the most rare being complete CA avulsion. To our knowledge, this case of CA avulsion from blunt polytrauma is only the 7th case reported in the literature. To assist in management, we discuss a case of blunt traumatic CA avulsion managed successfully with open ligation following endovascular balloon occlusion of the juxta-coeliac aorta for haemorrhage control.

## Case report

**Figure 1:**
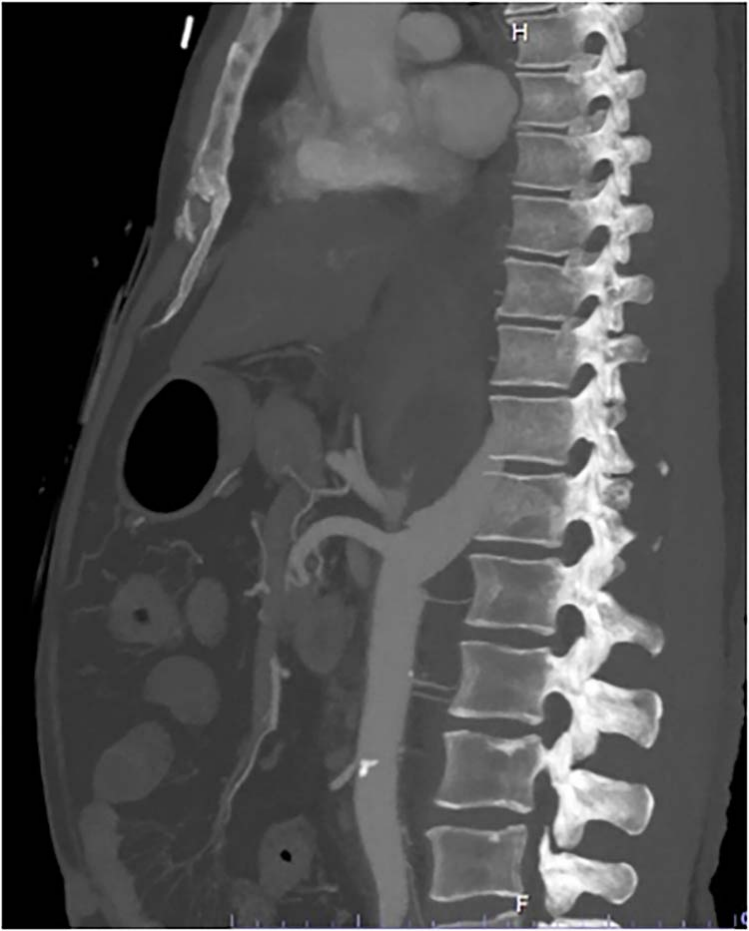
Sagittal CT slice demonstrating coeliac avulsion.

**Figure 2:**
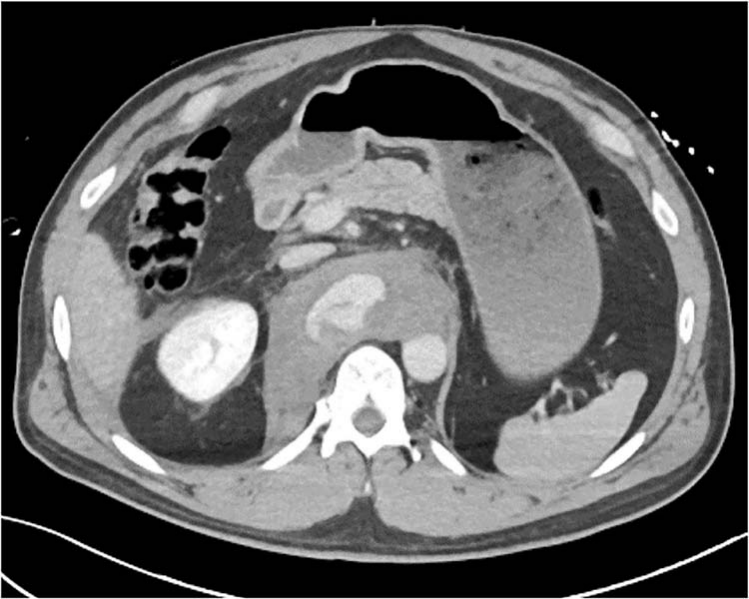
Axial CT slice demonstrating coeliac trunk avulsion with active bleeding into a large retroperitoneal haematoma.

**Figure 3:**
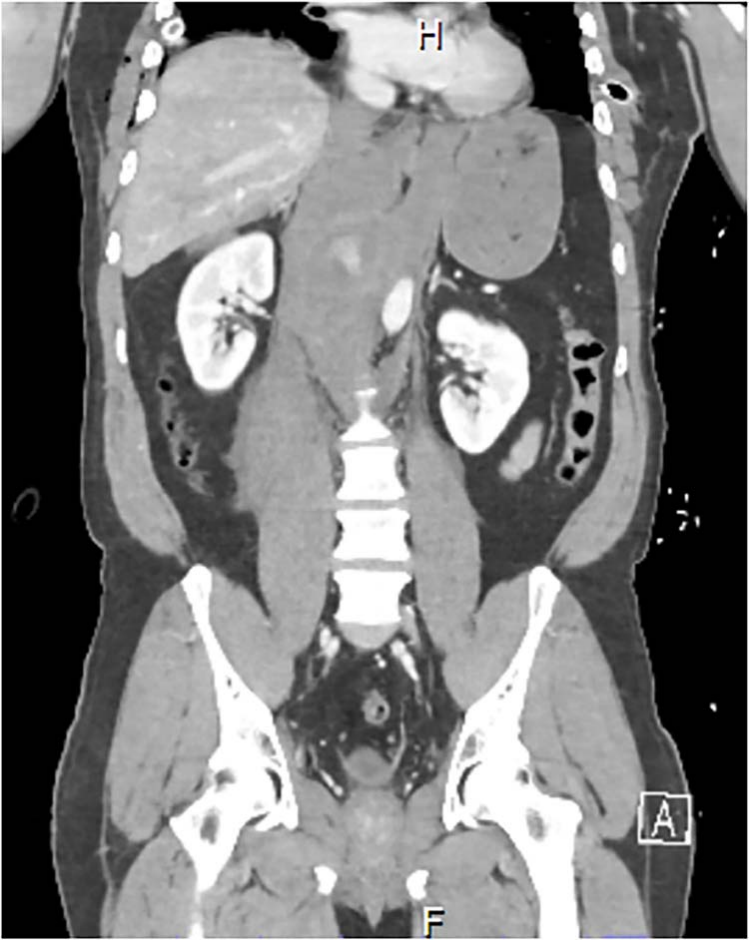
Coronal CT slice demonstrating coeliac trunk avulsion with active bleeding into a large retroperitoneal haematoma.

A 54-year-old male presented to our Level 1 Trauma Centre following a high-speed motor vehicle accident with rollover. Although initially haemodynamically stable, significant hypoxia prompted a trauma call activation. On arrival to the Trauma Centre, he had a Glasgow Coma Scale of 8 with an initial heart rate of 70 bpm and systolic blood pressure of 101 mmHg. A brief loss of cardiac output resulted in intubation, bilateral finger thoracostomies and the commencement of massive transfusion. Although his abdominal eFAST was positive, he was fluid responsive and proceeded to imaging. A full computed tomography (CT) trauma series demonstrated CA avulsion (Fig. 1) with contrast extravasation into the retroperitoneum and a large retroperitoneal haematoma measuring 18 cm by 9 cm (Figs 2 and 3). Nil visceral injury was identified and imaging otherwise revealed fractures of the left scapula, right 12th rib and bilateral acetabula and pubic rami.

**Figure 4:**
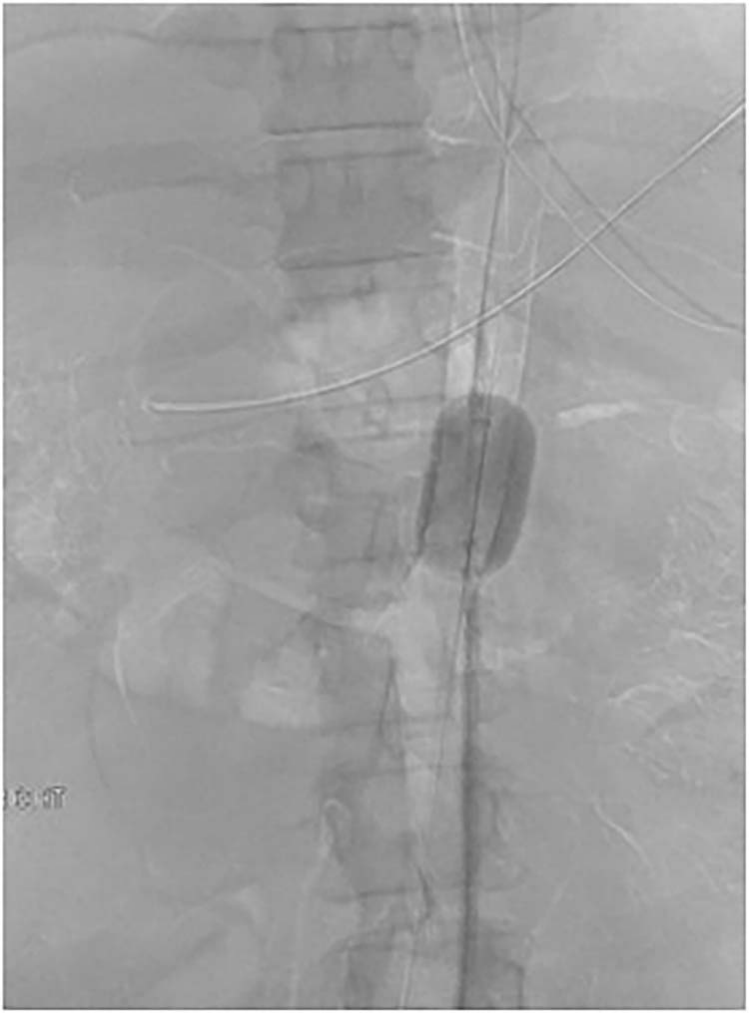
Fluoroscopic image demonstrating endovascular balloon occlusion of aorta at the level of CA avulsion, superior to SMA and Bilateral Renal artery origins.

The patient was emergently transferred to the hybrid theatre with Trauma Surgery and Vascular Surgery teams in attendance. Ultrasound-guided right common femoral artery retrograde access was obtained and catheter angiography confirmed CA avulsion with good retrograde filling of branch vessels from the superior mesenteric artery (SMA). A 32 mm Coda® compliant balloon (Cook Medical, Bloomington IN) [4] was introduced and held in place with a 12Fr 45 cm long DrySeal sheath (WL Gore, Flagstaff AZ) and positioned at the juxta-coeliac aorta for rapid hemorrhage control whilst a midline laparotomy was performed. A transperitoneal approach was taken to reach the supra-coeliac aorta and the endovascular balloon was then inflated to occlude the thoracic aorta, CA and SMA (Fig. 4). The distal and proximal ends of the CA were formally ligated with 3/0 polypropylene, with the trifurcation remaining in continuity. Completion angiography demonstrated forward flow into the SMA with retrograde filling of the common hepatic and splenic artery via the gastroduodenal artery (GDA). Temporary abdominal closure was performed with nil further visceral injuries demonstrated on a subsequent relook laparotomy prior to closure. He was discharged 47 days following his injury.

## Discussion

Blunt traumatic CA avulsions are rare and present a challenge to the most experienced of trauma teams. Due to the uncommon presentation, no grading system or guidelines for intervention exist [5]. Management is therefore dependent on a combination of the involved surgeon’s technical skills, operative resources, and access to specialty input. Of the 6 previous cases recorded in the literature (Table 1), four underwent open CA ligation [5–8], one underwent open aorto-coeliac bypass [9] and one underwent endovascular bridging with a stent [10]. At our Level 1 Trauma Centre, we performed a successful open CA ligation with the use of endovascular aortic control in the hybrid operating theatre with joint intervention by Trauma Surgery and Vascular Surgery teams.

**Table 1:** Cases of coeliac artery avulsion following literature review including age, mechanism, intervention, outcome and reference.

Age	Mechanism	Intervention	Outcome	Reference
17 M	MVC	Open ligation	Discharged	[9]
^a^	MBA	Open ligation	Death	[10]
58 M	MBA versus car	Open ligation	Discharged	[11]
72 M	MVC	Open ligation	Discharges	Osbourne *et al*., 2013 [8]
75 M	Pedestrian versus car	Open aorto-coeliac artery bypass	Discharged	[12]
39 M	Blunt trauma from steel bar	Endovascular bridging	Discharged	[6]

^a^Age unknown

The protected anatomy of the CA likely contributes to the rarity of this injury [11]. As the first major ventral branch of the aorta, the CA tracks inferiorly for 1–2 cm prior to splitting into three branches in 65–75% of people [12]. The close anatomical relationship of the CA to the median arcuate ligament (MAL) has been implicated in the pathogenesis of blunt trauma, resulting in a shearing effect of the MAL on the CA secondary to a sudden increase in intrabdominal pressure [8, 10]. Given the majority of cases reviewed suffered high velocity motor vehicle and motorbike accidents (Table 1) along with rotational forces from a full rollover in this case report, these mechanisms leading to CA avulsion are consistent with this theory.

Treatment options for CA avulsion include open repair with either lateral suture repair, bypass or ligation, endovascular repair with stenting or embolization, or a hybrid technique, particularly with increasing access to hybrid operating theatres. Open ligation is the simplest method and yields expedient results, particularly relevant in haemodynamically compromised patients. Due to the rich collateral supply of the mesenteric circulation, in particular by virtue of the GDA, few ischaemic complications have been encountered from this technique [3], although rare cases of gallbladder [13] and liver ischaemia [9] have occurred. As described in this case report, catheter angiography can be used to assess collateral supply and in the case of high risk of ischaemia, aorto-coeliac bypass can be considered a safe alternative to ligation alone [9].

Only one case of CA avulsion has described the use of sole endovascular technique via bridging with a stent graft [10]. Alternatively, the use of an aortic stent graft covering the avulsed CA stump with concurrent embolization of the detached distal CA has been suggested as a separate endovascular technique [10]. The more common utility of endovascular intervention is the use of aortic balloon occlusion to control haemorrhage prior to open ligation, as with our case study. This technique has benefits of being able to be performed under local anaesthetic with rapid improvements to the patients’ haemodynamic status [14]. However, given the need for fluoroscopic guidance, this may not be a possible option in the unstable patient and traditional open techniques obtaining supra-coeliac aortic control should be employed.

## Conclusion

CA avulsions are a rare sequelae of blunt abdominal trauma, however with the use of modern endovascular techniques and multidisciplinary intervention, successful outcomes can be achieved. Multicenter data is required to produce consensus-based treatment guidelines for this high mortality injury.

## Data Availability

No data was required for this article given this is a case report.
